# Residual Humidity in Paraffin-Embedded Tissue Reduces Nucleic Acid Stability

**DOI:** 10.3390/ijms24098010

**Published:** 2023-04-28

**Authors:** Peter M. Abuja, Daniela Pabst, Benjamin Bourgeois, Martina Loibner, Christine Ulz, Iris Kufferath, Ulrike Fackelmann, Cornelia Stumptner, Rainer Kraemer, Tobias Madl, Kurt Zatloukal

**Affiliations:** 1 Diagnostic & Research Centre for Molecular Biomedicine, Institute of Pathology, Medical University of Graz, Neue Stiftingtalstrasse 6, 8010 Graz, Austria; 2 Gottfried Schatz Research Centre for Cell Signalling, Metabolism and Ageing, Molecular Biology and Biochemistry, Medical University of Graz, Neue Stiftingtalstrasse 6, 8010 Graz, Austria; 3 BioTechMed-Graz, 8010 Graz, Austria; 4 Berghof Products & Instruments GmbH, 72800 Eningen, Germany

**Keywords:** fixed tissue, nucleic acid quality, next-generation sequencing

## Abstract

Molecular diagnostics in healthcare relies increasingly on genomic and transcriptomic methodologies and requires appropriate tissue specimens from which nucleic acids (NA) of sufficiently high quality can be obtained. Besides the duration of ischemia and fixation type, NA quality depends on a variety of other pre-analytical parameters, such as storage conditions and duration. It has been discussed that the improper dehydration of tissue during processing influences the quality of NAs and the shelf life of fixed tissue. Here, we report on establishing a method for determining the amount of residual water in fixed, paraffin-embedded tissue (fixed by neutral buffered formalin or a non-crosslinking fixative) and its correlation to the performance of NAs in quantitative real-time polymerase chain reaction (qRT-PCR) analyses. The amount of residual water depended primarily on the fixative type and the dehydration protocol and, to a lesser extent, on storage conditions and time. Moreover, we found that these parameters were associated with the qRT-PCR performance of extracted NAs. Besides the cross-linking of NAs and the modification of nucleobases by formalin, the hydrolysis of NAs by residual water was found to contribute to reduced qRT-PCR performance. The negative effects of residual water on NA stability are not only important for the design and interpretation of research but must also be taken into account in clinical diagnostics where the reanalysis of archived tissue from a primary tumor may be required (e.g., after disease recurrence). We conclude that improving the shelf life of fixed tissue requires meticulous dehydration and dry storage to minimize the degradative influence of residual water on NAs.

## 1. Introduction

Diagnostics in healthcare increasingly relies on the detailed molecular analyses of alterations of the genome and transcriptome using tissue specimens harvested during surgery or biopsy. Such analyses have become invaluable for diagnosis, therapy selection, and eventually personalized patient treatment. Quantitative real-time polymerase chain reaction(qRT-PCR)-based molecular diagnostics and gene-panel-based Next Generation sequencing (NGS) are readily established in cancer diagnostics, and due to the lowering of costs, whole-genome and whole-exome sequencing are increasingly used not only for research but also for diagnostics, which, however, places increasing demands on sample quality.

Tissue used for diagnostic purposes is usually formalin-fixed, paraffin-embedded (FFPE), where the fixative is 10% neutral buffered formalin (NBF, 4% formaldehyde *v*/*v*. in an aqueous phosphate-buffered solution). This material is widely available and has been used for decades not only for pathomorphological characterization but also for immunohistochemical, histochemical, and in-situ hybridization methodologies [1]. The use of FFPE tissue for transcriptomic and genomic analyses, however, revealed severe shortcomings regarding the quality and reliability of the results, in particular after prolonged storage at room temperature, showing a time-dependent aging effect [2,3,4,5]. This is a result of chemical modification of biomolecules (e.g., by crosslinking of nucleic acids (NA) and proteins) [6,7] or by modification of nucleobases [8,9,10]. Alternative, non-modifying fixatives, such as PAXGene^®^ Tissue System (PFPE), have been shown to give much better results and a longer shelf life [11]; however, they are not routinely used in healthcare.

Both qRT-PCR-based and advanced analysis methodologies, such as next generation sequencing, require input material in which NAs are sufficiently well preserved to yield reliable results [5]: Failing to detect a critical variant may invalidate the diagnosis with dire consequences for the patient’s treatment. Consequentially, there is growing emphasis on the quality of biospecimens, in particular their suitability for the intended diagnostic purpose. It has long been recognized that the critical steps in producing highly reliable results often do not lie with the analysis itself but rather with the pre-analytical workflow. Accordingly, a series of international standards for the pre-analytical processing of biological samples were developed [12,13,14,15].

Depending on the pre-analytical processing of fixed tissue, the quality of NAs and their shelf life may vary considerably, both for chemically modifying and non-modifying fixatives. Specifically, for FFPE tissue, a variety of parameters were described as impairing morphological representation and immunoreactivity [1,2,16]. Besides ischemia times during tissue harvesting (during and after surgery), fixative type, fixation temperature and duration, and the ratio of fixative and tissue volumes are important determinants affecting NA quality and shelf life [4].

The preparation of fixed tissue is a multi-step process. After immersing tissue into the fixative (at room temperature) for the appropriate time (‘fixation’), the material is dehydrated in a series of ethanol baths with increasing concentration (ending with 100%, which is usually 99% plus 1% denaturing agent), followed by replacing ethanol with a nonpolar solvent (‘clearing’) and finally the infiltration of tissue with paraffin (‘impregnation’) [17]. For PFPE tissue, only the ‘fixation’ step is different from FFPE tissue. 

For a long time, it was suspected that the quality of NAs and their stability are not only affected by the fixation process but also by the subsequent steps: residual water, resulting from improper dehydration after fixation, or from improper storage, may revert the fixation process [8] and lead to the hydrolytic degradation of NAs. Moreover, it can eventually lead to inhomogeneities in paraffin infiltration [18], which can render the sample more prone to degradation. Improper dehydration might be a consequence of the carryover of water along the ethanol series, which may increase the water content of the last bath. This may be caused, e.g., by the prolonged use of ethanol (often to reduce the cost), especially in manual dehydration protocols. It should also be noted that ethanol forms an azeotropic mixture with water (3.5% water), which can form upon the exposure of absolute ethanol to (humid) air. The experimental conditions we selected (95% and 90% ethanol) reflect these possibilities. Another factor is that fixed tissue can accumulate water from the environment over time; therefore, we also investigated changes in residual humidity during different storage scenarios. Finally, FFPE and PFPE are not only different by their fixation chemistry (cross-linking and non-cross-linking) [19] but also by way of other chemical modifications; formalin introduces oxidative modifications in the nucleobases of both RNA and DNA [20], such as cytosine deamination [21] and the sugar-phosphate backbone [22,23], which make the material more polar, facilitating the uptake and retention of water. The hydrolysis of the phosphodiester bond of DNA is quite slow compared to that of RNA [20,24], which contributes to the well-known fragility of RNA in fixed tissue. A detailed description of the alterations to nucleic acids by formalin and the consequences for NGS of FFPE material is given in [25]. 

It has been reported that the hydrolysis and alterations of nucleobases in mRNA have a deleterious influence on the outcome of qRT-PCR, making reliable quantitation impossible [5], which additionally compromises RNA-based diagnostics. Moreover, the modification of nucleobases, e.g., Cytidine deamination, can also lead to sequencing artifacts in diagnostic NGS [21,22,25], leading to unreliable diagnoses and compromised patient stratification.

In this study, we combined the measurement of temperature-dependent water release from FFPE and PFPE tissue (see Material and Methods, and Figure S1) with amplicon-length qRT-PCR and the nuclear magnetic resonance (NMR) spectroscopic analysis of the NAs to establish the causal link between residual humidity and reduced shelf life and compromised NA quality.

## 2. Results

### 2.1. Parameters Affecting Residual Humidity (RsH) and Amplicon-Length-Dependent qRT-PCR Performance Index (AL-PPI)

Residual humidity (RsH), i.e., the percentage of water remaining bound to tissue after dehydration), may be a major determinant of tissue NA quality. To unravel this complex interplay, we first showed how RsH is influenced by the fixation type. We then investigated the effects of the dehydration protocol as well as storage conditions and duration and how RsH relates to the amplicon-length qRT-PCR performance index (AL-PPI; see Materials and Methods below), reflecting the difference in the amplification efficiencies of short and long amplicons (the lower the AL-PPI, the more the RNA was modified and degraded). This was followed by a comprehensive analysis of all factors affecting the quality of NAs in fixed tissue. Note that although AL-PPI is derived from qRT-PCR results, we included amplicon lengths of up to 526 base pairs in its definition and measurement to account for the fact that sometimes read-lengths in NGS are much higher than those in qRT-PCR. Moreover, this allows a more finely graded assessment of the differences.

### 2.2. Influence of Fixation Type (at 100% EtOH) on RsH and AL-PPI

**Figure 1:**
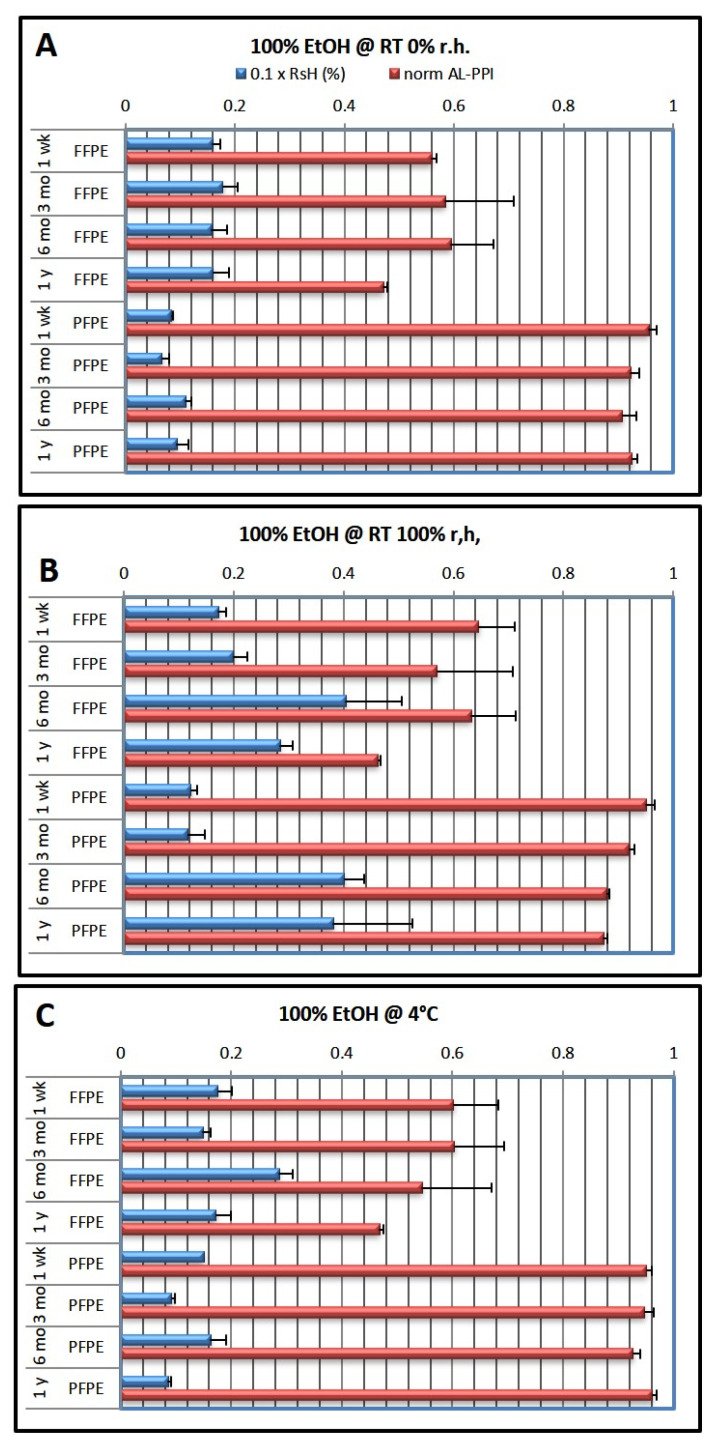
Effects of fixation type and storage conditions over time using the 100% ethanol dehydration protocol. The last ethanol step in the dehydration protocol was 100% ethanol (cf. Table 1). Blue columns show residual humidity (0.1 × RsH, wt.%; factor 0.1 chosen to allow one axis for both parameters), red columns show normalized qRT-PCR performance (AL-PPI; see Methods). (**A**) storage at RT and 0% r.h.; (**B**) storage at RT and 100% r.h.; (**C**) storage at 4 °C. Data are shown as mean ± standard deviation (n = 3 per condition).

In the first experimental series (Figure 1), the last EtOH dehydration step was always performed with 100% EtOH (optimal dehydration). 

After 1 week of storage, RsH was generally lower for PFPE than for FFPE samples (RsH_PFPE_ = 0.6–4%; RsH_FFPE_ = 1.5–4%). Prolonged storage for up to one year revealed that high ambient humidity (emulated by storage at room temperature (RT) and 100% relative humidity (r.h.) in a humid chamber) led to an increase in RsH, while storage at an ambient temperature in a dry atmosphere or at 4 °C left RsH largely unchanged over time. Interestingly, we found the increase in RsH at 6 months most pronounced both for FFPE and PFPE when stored at RT and 100% r.h. (RsH = 4%). 

The AL-PPI of PFPE tissue RNA was generally high and close to that of cryopreserved tissue, which served as a reference (AL-PPI_PFPE_ = 0.88–0.95; data were normalized so that AL-PPI_cryo_ = 1.0) and did not appreciably vary with RsH (except for a slight dip after 6 months of storage). Conversely, AL-PPI_FFPE_ was much lower, approximately half of that of PFPE or cryopreserved material (AL-PPI_FFPE_ = 0.43–0.64), with increased loss of qRT-PCR performance over time.

### 2.3. Dependence of RsH and AL-PPI on Dehydration Protocol

**Figure 2:**
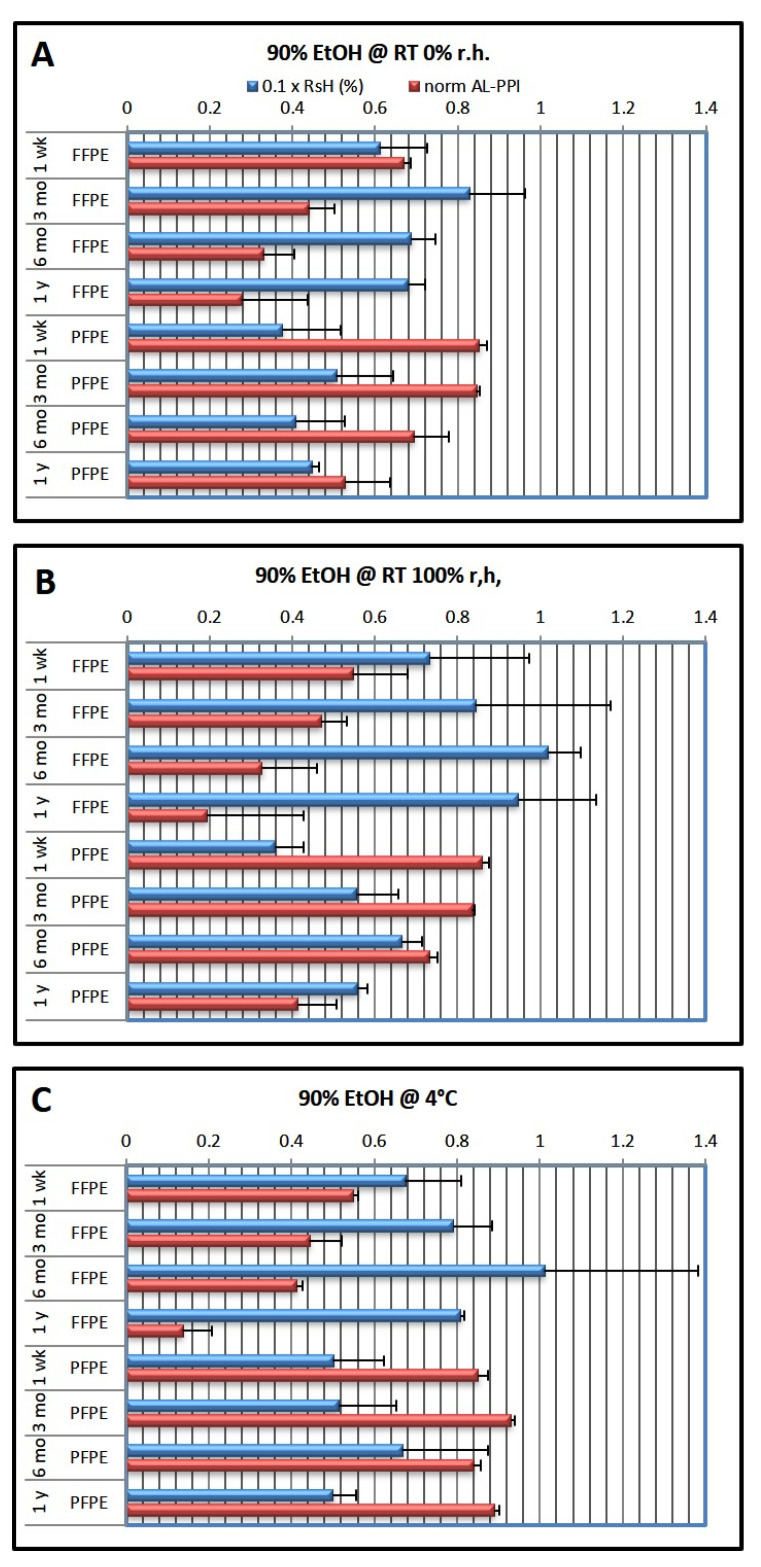
Effects of fixation type and storage conditions over time using the 90% ethanol dehydration protocol. The last ethanol step in the dehydration protocol was 90% ethanol (cf. Table 1). Blue columns show residual humidity (0.1 × RsH, wt.%; factor 0.1 chosen to allow one axis for both parameters), red columns show normalized qRT-PCR performance (AL-PPI; see Methods). (**A**) storage at RT and 0% r.h.; (**B**) storage at RT and 100% r.h.; (**C**) storage at 4 °C. Data are shown as mean ± standard deviation (n = 3 per condition).

In the second experimental series (Figure 2), the last EtOH dehydration step was always performed with 90% EtOH. Results showed that after 1 week of storage, RsH was much higher than after optimal dehydration, both for FFPE (RsH_FFPE_ = 6.1–10.0%) and PFPE (RsH_PFPE_ = 3.6–6.7%). FFPE tissue thus contained about twice as much residual water as PFPE tissue. RsH remained little changed at RT in a dry atmosphere (0% r.h.) but increased over time both at 100% r.h. at RT and at 4 °C, both for FFPE and PFPE tissue. Interestingly, a maximum of RsH could be observed after 6 months of storage under these conditions.

AL-PPI_FFPE_ decreased significantly over time, from 0.54–0.67 at 1 week to 0.13–0.28, nearly independent of storage conditions, and also between 6 months and 1 year of storage, where a decrease in RsH was observed, suggesting consumption of water through the hydrolysis of tissue macromolecules or the reversal of fixation [9]. 

AL-PPI_PFPE_ also decreased over time whenever tissue was stored at ambient temperature, from 0.85–0.86 to 0.41–0.53. This was not observed in PFPE tissue stored at 4 °C, where AL-PPI_PFPE_ remained between 0.85 and 0.93.

**Figure 3:**
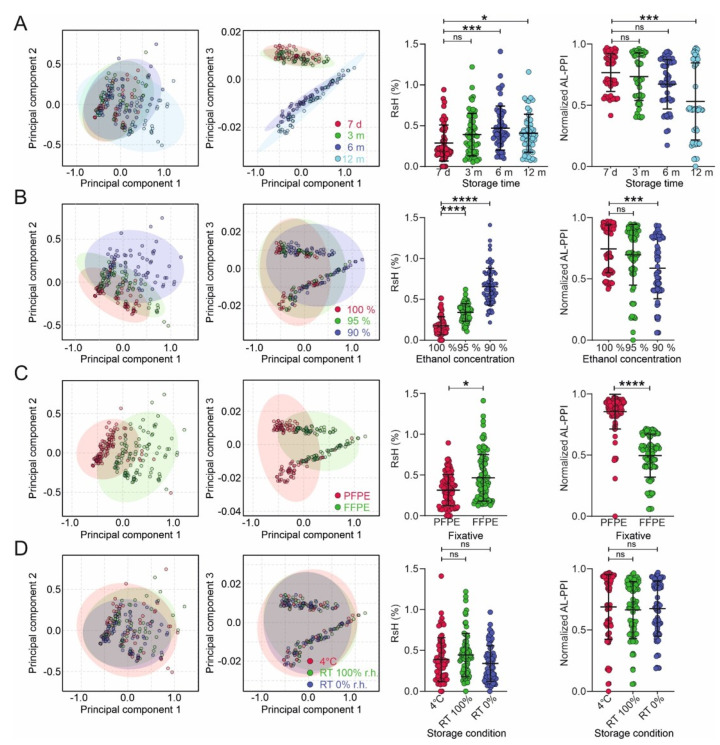
Multivariate analysis of humidity and qRT-PCR performance. Principal component analyses (PCA) plots of residual humidity (RsH) using storage time (**A**), ethanol concentration, (**B**), fixation method (**C**), and storage temperature/humidity as groups (**D**). A univariate statistical analysis of RsH and normalized PCR performance index (AL-PPI; see Supplementary Methods) as a function of the groups described in (**A**–**D**) is shown next to the PCA plots. Data are represented as mean ± standard deviation (SD). Statistical significance of the differences among multiple groups was determined using one-way ANOVA (Dunnett’s multiple comparisons test) or unpaired *t*-test for 2 groups. *p* > 0.05 = ns; *p* < 0.05 = *; *p* < 0.001 = ***; *p* < 0.0001 = ****.

To better describe the complex interactions of tissue fixation, dehydration, and storage protocols, we used the multivariate data analysis of RsH and AL-PPI. Principal Component (PC) Analysis (PCA) of RsH and (orthogonal) Partial Least Squares-Discriminant Analysis ((O)PLS-DA) of both parameters showed that RsH and AL-PPI are predominantly affected by storage duration, dehydration protocol, and fixation protocol. The temperature and humidity of storage did not significantly affect RsH, which is in line with the comparable qRT-PCR performance (AL-PPI) for samples stored under these conditions (Figure 3).

Storage duration (Figure 3A): Principal component (PC) analysis (PCA) (PC1 and PC3) shows a very clear separation of the short- and long-term stored samples—these PCs are clearly attributable to changes in RsH with storage duration. This is corroborated by OPLS-DA, showing that RsH increases significantly after 6 and 12 months of storage and that AL-PPI is significantly reduced after 12 months of storage.

Dehydration protocol (Figure 3B): PCA analysis shows no distinct separation of the different dehydration protocols; however, PC1 and PC2 show a distinct broadening of the RsH data with decreasing ethanol concentration in the last dehydration step (90% > 95% > 100%). OPLS-DA reveals highly significant differences between the RsH and AL-PPI values for the protocols.

Fixation type (Figure 3C): PCA reveals good separation for all shown PCs, and a distinctly broader distribution of RsH for FFPE samples (PC1 vs. PC2). OPLS-DA shows significantly higher RsH and significantly lower AL-PPI for FFPE compared to PFPE material.

Storage conditions (Figure 3D) have no significant influence on RsH and AL-PPI; however, dry storage at room temperature shows a narrower distribution of RsH data (PC1 vs. PC2) than the other storage conditions.

We also performed univariate analyses for the different conditions separately for FFPE and PFPE tissue (Supplementary Figure S3) with similar results.

### 2.4. NMR Results

Given the impact of tissue fixation protocols on qRT-PCR performance, we hypothesized that (R)NA accessibility and modifications are causing these differences. To obtain insight into potential NA modifications in extracted samples, we used untargeted metabolomic analysis via NMR spectroscopy. Extracted DNA and RNA are large polymers and are difficult to study via solution NMR. To overcome this limitation and obtain single nucleotides, we performed the complete enzymatic digestion of nucleic acids using a mix of alkaline phosphatase, benzonase, and phosphodiesterase. 

The 1D ^1^H-NMR analysis of the digested nucleic acid samples showed the presence of several NMR signals characteristic of (modified) nucleotides and sugars at different concentrations. The assignment of these signals revealed adenosine, cytidine, guanosine, and uridine as the main digestion products. In a subset of samples, additional ribose amplicons, 2′-deoxyadenosine, 2′-deoxycytidine, 2′-deoxyguanosine, thymidine, and base degradation products were detected (Supplementary Figure S4).

**Figure 4:**
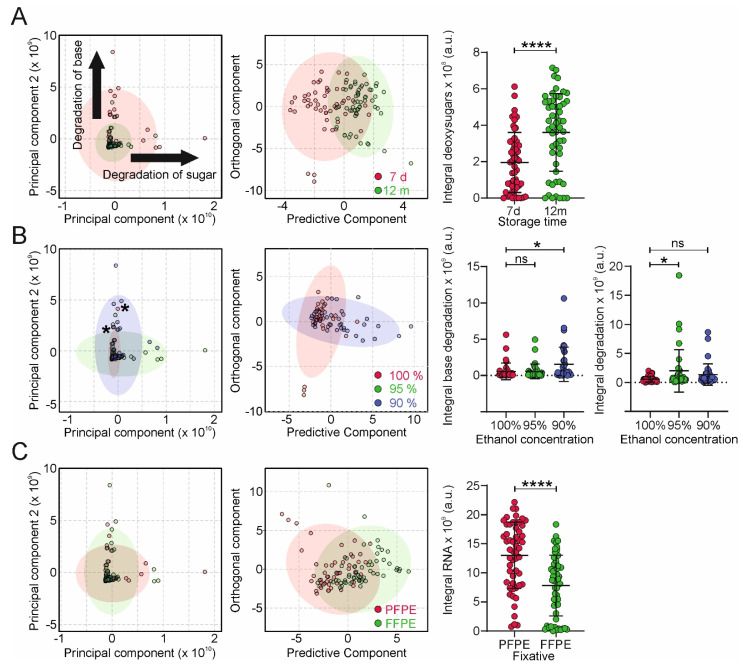
Multivariate NMR-based analysis of digested nucleotide extracts. Principal component analyses (PCA, left two panels), partial least squares-discriminant analysis (PLS-DA, right panels, >2 groups) and orthogonal PLS-DA (right panels, 2 groups) plots of NMR samples using storage time (**A**), ethanol concentration (**B**), and fixation method (**C**) as groups. A univariate statistical analysis of normalized metabolite group concentrations associated with significant changes observed in the O-PLS-DA analysis as a function of the groups described in (**A**–**C**) is shown next to the PCA/PLS-DA/O-PLS-DA plots. Data are represented as mean ± standard deviation (SD). Statistical significance of the differences among multiple groups was determined using one-way ANOVA (Dunnett’s multiple comparisons test) or unpaired *t*-test for 2 groups. *p* > 0.05 = ns; *p* < 0.05 = *; *p* < 0.0001 = ****.

To characterize the impact of tissue fixation and storage protocols on metabolite distribution, we used PCA and (O)PLS-DA. Similar to RsH and AL-PPI, the metabolite profiles were mainly affected by storage duration, residual water, and the fixation protocol. (Figure 4). 

Storage for 12 months revealed increased heterogeneity (Figure 4A), indicated by a broader distribution over PC1 and PC2. We observed large changes in two principal components, indicating degradation of the sugar and base moieties, respectively. OPLS-The DA analysis of NMR-based metabolite profiles using storage duration as groups revealed clustering with correlation coefficients R^2^Y of 0.519 and Q^2^ of 0.351 (*p* < 0.01; Figure 4A). The inspection of the OPLS-DA loading profiles revealed 2′-deoxyadenosine, 2′-deoxycytidine, 2′-deoxyguanosine, and thymidine as significantly increased metabolites. We then compared the impact of the dehydration protocol on the NMR metabolite profiles. As shown in Figure 4B, increased concentrations of residual water led to more heterogeneous metabolite profiles. The OPLS-DA analysis of NMR-based metabolic profiles using 90% and 100% EtOH as groups revealed base and sugar degradation products as significantly increased degradation products in FFPE tissue (R^2^Y = 0.226, Q^2^ = 0.067, *p* = 0.02; Figure 4B). In addition to storage duration and dehydration protocol, the fixation type has an impact on metabolomic profiles. Using OPLS-DA analysis, we identified decreased concentrations of adenosine, cytidine, guanosine, and uridine in FFPE samples (R^2^Y = 0.371, Q^2^ = 0.310, *p* < 0.01; Figure 4C). This can be explained by the significantly reduced nucleic acid yield in FFPE tissue after hydrolysis, compared to PFPE, due to increased cross-linking of nucleic acids, resulting in lower accessibility for the enzymes. 

## 3. Discussion 

In this study, we have demonstrated that the type of tissue fixation, the amount of residual water, storage conditions, and the duration of tissue storage impact the quality of NA (DNA, RNA) in tissue. Residual water content is affected by the type of fixation (cross-linking, non-cross-linking) and by the reaction of formalin with NAs, making them more polar and favoring increased water uptake and retention. Further influencing factors are the fixation protocol (100%, 95%, 90% final EtOH step) and storage conditions (humid, dry, 4 °C). Residual water in turn correlates with the quality of RNA, regarding the performance in qRT-PCR, and the stability of nucleic acids, as shown by the hydrolysis products in the NMR analysis. Apart from the fixation type, which affects NA quality the most, residual humidity is the most prominent factor leading to NA degradation in stored samples.

Fixation with formalin is known to dramatically impair the quality of NA for Next-Generation sequencing [20,26] but also for qRT-PCR methods. Formalin oxidatively modifies NA, specifically the nucleobases [20,21,22] of both DNA and RNA, which leads to sequencing artefacts and the reduced performance of qRT-PCR-based techniques [27]. We have observed this also in our NMR experiments (Figure 4C), where the yield of ribonucleotides is substantially lower in FFPE compared to PFPE tissue, corroborating the notion of the sensitivity of RNA and DNA nucleobases towards modification by formalin [20]. On the other hand, DNA is comparatively resistant regarding hydrolysis of the phosphodiester bond [20], unlike RNA, where it is more easily cleaved by nucleophilic attack by the deprotonated 2′OH group of deoxyribose [24]. Moreover, it leads to cross-linking of NAs with polynucleotides [9,19] and polypeptides [7], resulting the in reduced availability of NAs for analysis, which is reflected in the lower yield in the NA digestion for NMR analysis. In addition to that, our results also show that FFPE tissue contains a high content of residual humidity due to the higher polarity of the modified NAs and amino acids and is more hydrophilic during storage in a non-dry environment. On the other hand, non-cross-linking fixation methods, such as PAXgene^®^ TissueFix used in this study, do not modify NAs and amino acids, leading to lower residual humidity and lower hydrophilicity, resulting in the better stability of NAs. 

This highlights the importance of a robust tissue processing protocol in which the residual water content is as low as possible, specifically for achieving long-term stability of NAs. Such a protocol features an ethanol dehydration series that concludes with 100% ethanol before the dehydrated tissue is transferred to the clearance step (usually immersion in xylene) that allows the final paraffin infiltration to proceed evenly and completely. Incomplete paraffin infiltration was shown to compromise the quality of the final, embedded tissue [18]. In many routine pathology laboratories, such protocols are well established, usually by automated tissue processing machines, and all dehydration and clearing bath solutions are regularly replaced to prevent water from accumulating in the final (100%) EtOH bath(s). However, we show here the possible consequences and the dangers that may occur with manual tissue processing, where solvent baths may be changed less regularly (e.g., due to economic pressure or impaired supply chains) or become exposed to air humidity for a long time, leading to the well-known EtOH/water azeotrope.

Residual humidity drastically reduced the performance of qRT-PCR, in particular for the FFPE tissue. From a previous study, we know that this is mainly due to the compromised performance of the reverse transcriptase reaction [5]. By comparison, the PFPE tissue generally contained less residual water and showed a better qRT-PCR performance. The main reason is that PFPE tissue is not chemically modified, as is the case with formalin. In PFPE tissue, the hydration shells of protein and nucleic acid macromolecules are altered, which leads to coagulation in situ. This per se does not introduce chemical modifications that may increase the polarity of the side chains of amino acids and nucleobases and is the active principle of PAXGene^®^ Tissue Fix and other agents that act via coagulation using water-miscible alcohols and organic acids. Formalin, on the other hand, does not only lead to cross-linking; it also increases hydrophilicity via chemical modification [8,9]. Moreover, the major modifications that have been found previously in FFPE tissue (such as the hydroxymethylation of nucleobases [9,25]) are reversible via hydrolysis, and the reactive products formed in this process may lead to further modifications over time. We could not detect specific differences between RNA and DNA nucleobases or their hydroxymethyl derivatives via NMR in our experiments.

Owing to the specific modifications of the nucleic acids by formalin, the effect of storage time is particularly pronounced for FFPE tissue after 6 months, while the storage conditions (i.e., temperature and humidity) have little effect. Nucleic acids in PFPE tissue are generally more stable than in FFPE material, and they are less affected by storage time, where we see pronounced alterations later. Those from FFPE tissue seem to benefit a little from dry storage at room temperature. Overall, PFPE tissue preserves a much better quality of NAs that are more stable and yield better than FFPE material. 

Comprehensive metabolomics data provide new insights into the mechanistic understanding of the link between tissue fixation protocols and sample quality (using qRT-PCR as the readout). The metabolomic characterization of digested NA samples obtained from differently prepared tissues revealed relevant metabolite markers for sample quality. Our analysis revealed alterations in NA yield, NA dehydration, sugar, and base modification in extracted DNA samples. Moreover, we found that the tissue fixation type affects the metabolite profile of digested NA samples and thus their degradation profiles. The related metabolites might be used and further extended in follow-up studies as markers in clinical settings for the quality control of fixed tissues and to optimize fixation protocols, which is a difficult task when relying on qRT-PCR alone.

Using the residual water measurement principle based on a P_2_O_5_ sensor, we could demonstrate in this study the correlation of residual water on NA stability in stored tissue samples, which can be explained by the fact that even under optimal conditions, not all the water is removed during tissue processing. A small fraction of water remains bound to membranes, NAs, and proteins as an integral part of the secondary and tertiary structures that cannot be removed without collapsing them. Indeed, removing this ‘structural water’ requires comparatively harsh methods, such as heating to temperatures that may already lead to the chemical decomposition of the molecules. However, it is unlikely that structural water participates in widespread degradation processes since it is strictly localized. In addition, water forms a hydration shell around biological macromolecules and assemblies, such as cell membranes. In addition, there is interstitial water (extracellular), which is even more mobile and can partake in hydrolysis reactions, similar to water in the hydration shell. These types of water are largely removed during tissue processing, completing the precipitation process that was initiated by the fixative, and allowing infiltration of the hydrophobic paraffin. In native tissue, more water is not directly engaged in hydrating macromolecules (often not quite correctly termed ‘bulk water’) but contributes to the mobility of the macromolecules and participates in exchanges with the hydration shell. Bulk water, too, is removed during tissue processing. During storage, water from the surrounding air is in equilibrium with the paraffinized tissue (there is likely exchange through micropores and cracks), becomes loosely adsorbed, and is in equilibrium with the residual water. That means that residual water in tissue may be composed of structural water, the hydration shell, and bulk water catalyzing the hydrolytic degradation of NAs and other biomolecules.

Based on our results, we may give recommendations for the preparation of fixed tissue that contains high-quality NAs: (i) if possible, use non-crosslinking fixatives; (ii) ensure that the dehydration protocol is meticulously observed—specifically; the last dehydration step must not be compromised by >1% water in ethanol; (iii) store the samples as quickly as possible in a cool and dry atmosphere. Note that this includes all types of dehydrated, fixed, and embedded samples, e.g., cell blocks. Furthermore, our study emphasizes the importance of considering and documenting in detail fixation procedures, tissue processing, and the age of samples used in research projects. This is important since the integrated analysis of data generated from samples of different processing and ages may result in major biases in the results obtained. We also recommend normalizing study groups for the storage duration of biobanked or archived samples

Otherwise, in particular with FFPE tissue, setting up a study in which ‘young’ and ‘old’ samples are mixed and where dehydration protocols were not precisely followed or not comparable may lead to high variation in NA analyses and, in the worst case, wrong results. In NGS analyses, such samples will lead to a large dropout rate due to failed quality checks, favoring ‘young’ samples.

## 4. Materials and Methods

Absolute ethanol and xylene were from Merck (Vienna, Austria).

### 4.1. Experimental Design

After harvesting, tissue samples were fixed either with NBF or PAXGene Tissue Fixative^®^ (PGTS, PreAnalytiX GmbH, Hombrechtikon, Switzerland) and subsequently subjected to one of the dehydration/embedding protocols shown in Table 1 below. Embedded tissue blocks were then stored under different conditions (RT 0% r.h., 100% r.h., and 4 °C) for different periods of time (1 week, 3 months, 6 months, and 1 year).

While dehydration with 95% ethanol (EtOH) as the last dehydration bath already leads to significantly compromised NA quality and shelf life, we decided to reduce EtOH content even to 90% since the differences became more distinct—NMR measurements also benefited from the larger number of compromised NAs. For details of the experimental protocols, see Materials and Methods below. For clarity, we often show only the results for 100% (nominally) and 90% EtOH; Supplementary Data contain all data in a database.

### 4.2. Tissue Collection

We used mouse liver tissue to have proper control over pre-analytical parameters such as the interval between tissue harvesting and onset of fixation (cold ischemia). Furthermore, the liver is a large organ with a relatively homogenous composition that allows several aliquots from a single organ. Since no enzymatic activities are present in fixed tissue, only minor tissue-type-related differences in degradation are expected.

Mice (Swiss albino Him-OF-1) were kept on standard chow (Ssniff, Spezialdiäten GmbH, Soest, Germany) under identical standard conditions (controlled humidity and temperature, 12-hour day/night cycle) until sacrifice by cervical dislocation followed by liver harvesting within 1–2 min. Isolated livers were rinsed in ice-cold phosphate-buffered saline, and the lateral and medial lobes were grossly divided into three equally sized portions, which were immediately transferred to a labeled tissue cassette and immersed in the respective fixative. For reference, aliquots were snap-frozen in isopentane, precooled with liquid nitrogen, and stored in liquid nitrogen until use. The whole workflow required 4–5 min for each animal.

Organ harvesting does not require a specific animal experimentation license by Austrian law since approved conditions for the housing, feeding, and killing of the animals were in place (license for the facility BMWF-66.010/0147-V/3b/2018). 

### 4.3. Tissue Fixation, Processing, and Storage

After either 24 h of fixation in 10% neutral-buffered formalin (NBF; corresponding to 4% *v/v* formaldehyde in phosphate buffer) with a pH of 6.8–7.2 or 4 h in PGTS followed by 24 h of stabilization in PAXgene Tissue Stabilizer (PreAnalytiX, Hombrechtikon, Switzerland), the tissue was dehydrated in ethanol solutions, cleared in xylene, and impregnated with low-melting paraffin in a Microm STP 120 (Thermo Scientific, Vienna, Austria) tissue processor that was specifically reserved for this experiment; fresh solutions were prepared before processing the batches for a storage time-point. 

To avoid contaminating the dehydration reagents with formalin, NBF tissue was immersed in a separate 70% ethanol bath for at least 1 h prior to processing; processing was performed separately for NBF and PGTS tissue. 

The three protocols used for dehydration, clearing, and paraffin infiltration are shown in Table 1. They only differed in the ethanol concentration in the last two ethanol steps (7, 8) before clearing and infiltration with low-melting paraffin. After processing, the tissue was embedded completely in paraffin wax and stored for the appointed storage duration. For each storage duration (1 week, 3 months, 6 months, and 1 year), 3 batches of 18 samples each were processed on 3 consecutive days. For each of the batches, one of the final ethanol concentrations was used for steps 7 and 8, while the other steps (1–7 and 9–12) remained unchanged (54 samples were processed in total). 

After embedding, the FFPE and PFPE blocks were transferred to the appropriate storage environment. Blocks were kept in airtight polyethylene boxes either at room temperature (RT; monitored/controlled at 22–25 °C) or at 4 °C in the refrigerator, unopened until processing. Storage at RT was either under dry conditions (desiccant—MiniPax desiccant packages, Sigma Aldrich, Vienna, Austria) or at 100% (humid chamber and not in direct contact with liquid). Storage at 4 °C was performed without specific humidity control. Table 1 gives an overview of the storage conditions and duration. Since the storage boxes were not opened during the whole storage time, we did not observe mold formation even after one year of storage at 100% r.h.; however, it became noticeable under these conditions several months after opening.

### 4.4. Tissue Sectioning for Humidity Measurement and RNA Extraction

For humidity measurement, the paraffin wax surrounding the tissue was carefully removed and tissue blocks sectioned into 20 µm (nominal setting on the microtome; HM355/Histocom, Vienna, Austria) flakes as a compromise between sufficiently fast water diffusion during heating (see below) and rapid handling. Twenty-milligram flakes (approximately; precisely weighed) were placed into a 5 mL crimp-sealed vial with Teflon^®^-sealed stoppers immediately after sectioning and weighing (RotiLabo crimp-sealed, long-necked flasks, Roth, Vienna, Austria).

Immediately after sectioning for humidity measurement, 5–10 mg of 5 µm sections were prepared on the microtome for NA extraction. RNA was extracted using the RNeasy FFPE Kit (QIAGEN GmbH, Hilden, Germany) for FFPE, the PAXgene Tissue RNA Kit (PreAnalytiX, Hombrechtikon, Switzerland) for PFPE, or the Invitrogen TRIzol procedure (Life Technologies, Darmstadt, Germany) for snap-frozen tissue controls according to the manufacturer’s instructions.

The RNA concentration in the extracts was determined using a NanoDrop ND-1000 spectrophotometer (NanoDrop Technologies - ThermoFisher Scientific, Wilmington, DE, USA), and electropherograms were obtained using an Agilent 2100 Bioanalyzer platform with an Agilent RNA 6000 Nano Kit (Agilent Technologies, Santa Clara, CA, USA). Agilent 2100 Expert software version B.02.03.SI307 was used to calculate the RNA integrity number (RIN).

Additionally, total nucleic acids were extracted using the QIAamp™ DNA FFPE Tissue Kit (QIAGEN GmbH, Hilden, Germany) for FFPE and the PAXgene Tissue DNA Kit (PreAnalytiX) for PFPE, without RNAse digestion. The nucleic acid concentration in the extracts was determined using a NanoDrop ND-1000 spectrophotometer.

### 4.5. Humidity Measurement

Tissue humidity was measured with an instrument based on the EasyH_2_O™ humidity measurement instrument that was provided by Berghof GmbH (Eningen, Germany). It was modified to avoid clogging of the tubing and sensor by deposition of evaporated paraffin. The measurement principle is based on a P_2_O_5_ sensor [28], which detects water via the hydrolysis of P_2_O_5_ to metaphosphoric acid, H_2_PO_4_, and subsequent electrolysis of the water bound in this fashion, which regenerates P_2_O_5_. The charge required for electrolysis is directly proportional to the water bound to the sensor and was recorded as electrolysis current over time. The instrument performance and accuracy were checked before and after each measurement series (9–15 measurements) with apura^®^Water Standard Oven 1% (Na_2_WO_4_·2 H_2_O, Merck, Vienna, Austria; 15–25 mg; 1% *wt*/*wt* water content). Deviations from the nominal value were always below 2%. 

Water was released from the sample by raising the temperature, and water vapor was transferred to the sensor by means of a stream of pressurized air and dried by passing through a molecular sieve bed (10 Å beads, Roth, Karlsruhe, Germany, bed volume 1 L). The temperature was controlled by heating the vial with the sample in an air bath heated by a resistance heater. By controlling increase in temperature, it is possible to distinguish between loosely adsorbed (‘ambient’) water (released below 70° C) and water more tightly bound in the hydration shell (released above 100 °C) [29,30]. Water released was calculated as the percentage of the original tissue weight, including infiltrated paraffin (residual water, RsH). For a typical water release curve, see Supplementary Figure S2.

Humidity data were evaluated using Origin Pro 2.0 (Origin Labs, Friedrichsdorf, Germany), the specific curve fitting, and the extrapolation and integration functions. A detailed description of the data evaluation is provided in Supplementary Methods.

### 4.6. Fragment-Length-Dependent Quantitative RT-PCR on Mouse Liver

We have previously described an amplicon-length-dependent performance assay for qRT-PCR [5] in human tissue where we exploited the differences in the amplification of *GAPDH* to rate the RNA quality. This method allows a finer-graded assessment of RNA quality than the RNA integrity number does, in particular for FFPE tissue. However, the mouse genome contains several *Gapdh* iso- and pseudogenes, which precluded using *Gapdh* as a reference gene. Instead, we used the mouse gene *Ywhaz*, which appeared well-suited to the task [31,32], using five amplicons of lengths ranging from 84 to 526 bases. Details about the protocol are shown in the Supplement Materials, and primer sequences are shown in Supplementary Table S1. The fragment lengths selected in our assay were chosen so that the sensitivity to degradation was higher than originally used in [5]. We included amplicon lengths of up to 526 base pairs in its definition and measurement to account for the fact that sometimes read-lengths in NGS are much higher than in qRT-PCR. Moreover, this allows a more finely-graded assessment of the differences.

### 4.7. Evaluating Performance in an Amplicon-Length-Dependent qRT-PCR Assay—Amplicon-Length Performance Index (AL-PPI)

We and others have previously demonstrated that qRT-PCR is quite sensitive to RNA quality [5,11,33], which is reflected by increased C_q_ values for longer amplicons or even no result at all. Although these data give sufficient distinction between the quality of different NAs, they are difficult to directly use in statistical analyses. We have therefore empirically designed an amplicon-length qRT-PCR performance index (AL-PPI) that integrates the relation of C_q_ with amplicon length. Details on the calculation of this index are given in the Supplement Materials, and an illustration of the empirical match between this index and the amplicon-length qRT-PCR data is shown as Supplementary Figure S2.

### 4.8. Data Evaluation

PCR and humidity measurements were performed in triplicate for both humidity measurements and qRT-PCR performance. RsH and AL-PPI were the parameters used for evaluation. For each triplicate, the mean value and standard deviation were calculated to obtain a single parameter data point. 

### 4.9. NMR Sample Preparation, Data Acquisition, and Analysis

The digestion of NA (DNA and RNA) was necessary to make NAs accessible for NMR experiments, as in NA polymers, ^1^H signals are broadened beyond detection. The protocol is described in detail in Supplementary Methods. 

The NMR spectra of digested NA samples were recorded at 310 K using a Bruker Avance Neo 600 MHz NMR spectrometer (Bruker, Billerica, MA, USA) equipped with a TXI probe head. The Carr–Purcell–Meiboom–Gill (CPMG) pulse sequence was used to acquire ^1^H 1D NMR spectra with pre-saturation for water suppression (cpmgpr1d, 512 scans, 73,728 points in F1, 12,019.230 Hz spectral width, 1024 transients, and recycle delay of 4 s). NMR spectral data were processed as previously described [34]. The quantification of metabolites was carried out via the signal integration of NMR spectra. To account for different DNA concentrations in the extracts, integrals were normalized based on the amount of extracted DNA. Univariate statistical analysis was carried out using Graph Pad Prism 5.01. (GraphPad Software, La Jolla, CA, USA). Data are represented as mean ± standard deviation (SD). 

Statistical differences among multiple groups (one-way ANOVA) are indicated by *p*-values of < 0.05 (*), < 0.01 (**), or < 0.001 (***). A detailed description of the procedures is given in the Supplement Materials.

## Figures and Tables

**Table 1:** Tissue dehydration, clearing, and paraffin infiltration protocols used in this study.

Protocol:		100% Ethanol	90% Ethanol	95% Ethanol
Step	Time	Media		
1	15 min	70% Ethanol	70% Ethanol	70% Ethanol
2	15 min	70% Ethanol	70% Ethanol	70% Ethanol
3	30 min	80% Ethanol	80% Ethanol	80% Ethanol
4	1 h	90% Ethanol	90% Ethanol	90% Ethanol
5	1 h	96% Ethanol	96% Ethanol	96% Ethanol
6	1 h	96% Ethanol	96% Ethanol	96% Ethanol
7	1 h	100% Ethanol	90% Ethanol	95% Ethanol
8	1 h	100% Ethanol	90% Ethanol	95% Ethanol
9	1 h	Xylene	Xylene	Xylene
10	1 h	Xylene	Xylene	Xylene
11	1 h 30 min	Paraffin (55 °C)	Paraffin (55 °C)	Paraffin (55 °C)
12	1 h 30 min	Paraffin (55 °C)	Paraffin (55 °C)	Paraffin (55 °C)

## Data Availability

All data used in this study appear in the paper’s text, figures, and supplementary material.

## Supplementary Materials

The supporting information can be downloaded at: https://www.mdpi.com/article/10.3390/ijms24098010/s1. References [34,35] were cited in supplementary materials.
