# Supplementary material for: Residual Humidity in Paraffin-Embedded Tissue Reduces Nucleic Acid Stability
Source: Int J Mol Sci. 2023 Apr 28;24(9):8010. doi: 10.3390/ijms24098010 (PMC10178321; doi:10.3390/ijms24098010)

## Slide 1
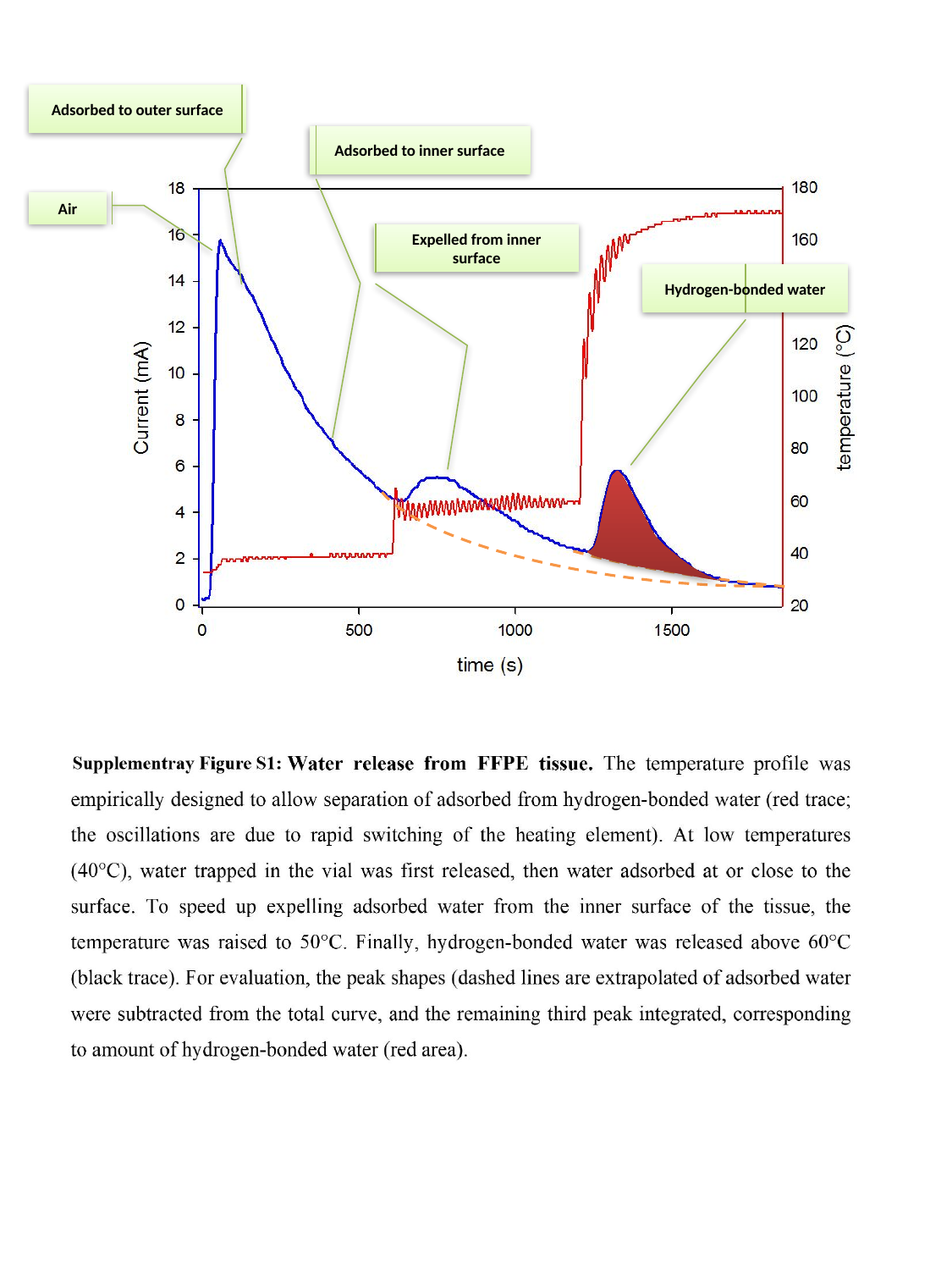

Adsorbed to outer surface
Adsorbed to inner surface
Air
Expelled from inner surface
Hydrogen-bonded water

## Slide 2
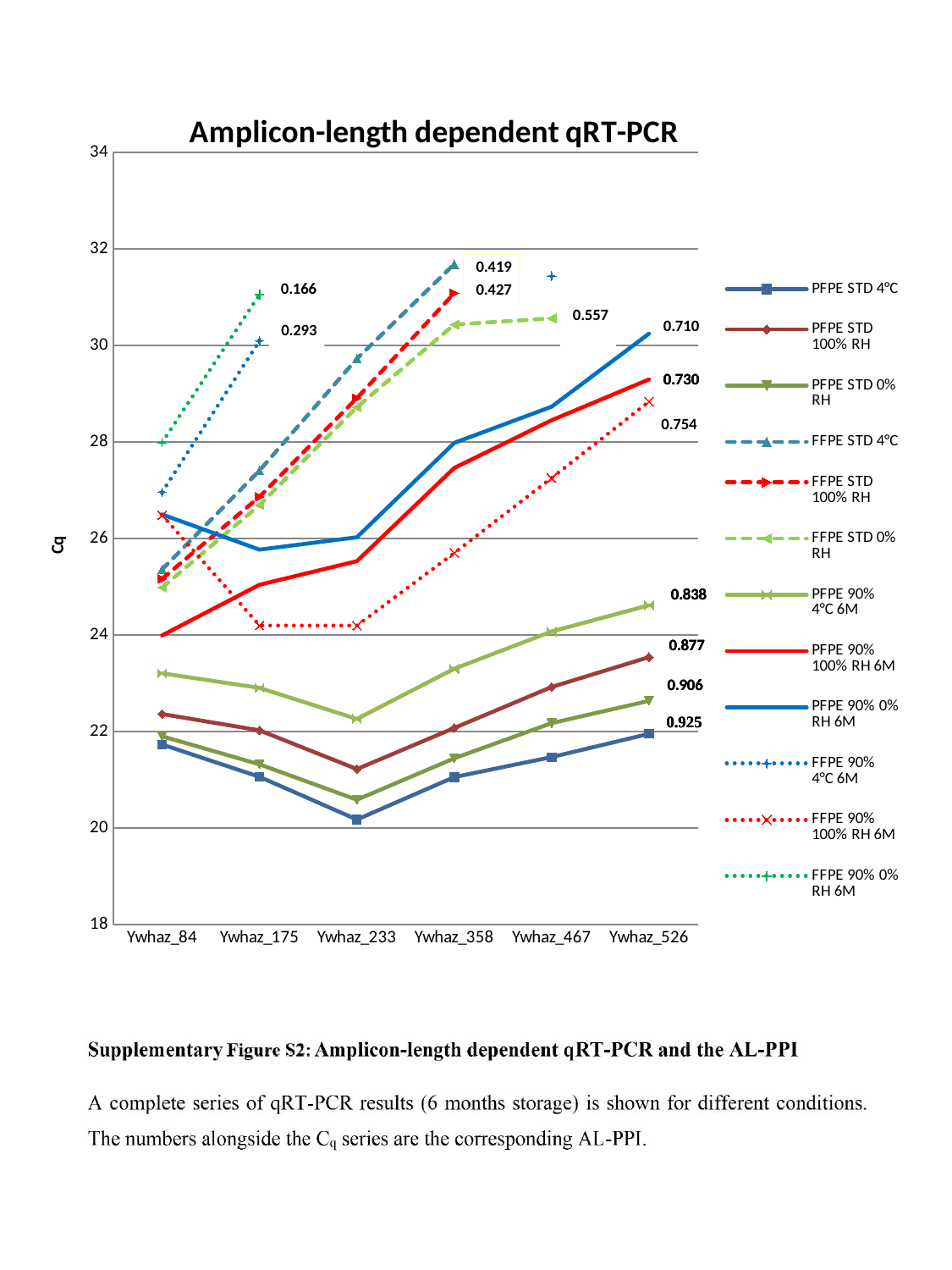

### Chart: Amplicon-length dependent qRT-PCR
| Category | PFPE STD 4°C | PFPE STD 100% RH | PFPE STD 0% RH | FFPE STD 4°C | FFPE STD 100% RH | FFPE STD 0% RH | PFPE 90% 4°C 6M | PFPE 90% 100% RH 6M | PFPE 90% 0% RH 6M | FFPE 90% 4°C 6M | FFPE 90% 100% RH 6M | FFPE 90% 0% RH 6M |
|---|---|---|---|---|---|---|---|---|---|---|---|---|
| Ywhaz_84 | 21.732767740885418 | 22.36317210727268 | 21.903785069783527 | 25.36004490322537 | 25.165134853786892 | 24.984352959526905 | 23.207436031765408 | 23.992549684312607 | 26.498844570583767 | 26.96264245775011 | 26.48670366075304 | 27.989653905232746 |
| Ywhaz_175 | 21.06284523010254 | 22.025561438666447 | 21.321287790934246 | 27.40979067484538 | 26.864758385552303 | 26.69360054863824 | 22.904525968763565 | 25.042011260986328 | 25.769001007080078 | 30.096972571478947 | 24.197612126668293 | 31.052757051255966 |
| Ywhaz_233 | 20.17772038777669 | 21.22289827134874 | 20.588071823120117 | 29.72607962290446 | 28.909192827012802 | 28.72274653116862 | 22.26174481709798 | 25.53121778700087 | 26.02566623687744 | None | 24.19593874613444 | None |
| Ywhaz_358 | 21.057380040486652 | 22.07393731011285 | 21.44629118177626 | 31.685299237569172 | 31.084163029988606 | 30.43366845448812 | 23.301685545179577 | 27.46156374613444 | 27.98117478688558 | None | 25.699373881022137 | None |
| Ywhaz_467 | 21.471805148654514 | 22.92324299282498 | 22.175019794040256 | None | None | 30.560961723327637 | 24.07341363694933 | 28.450484911600746 | 28.73189353942871 | 31.436779022216797 | 27.254421869913738 | None |
| Ywhaz_526 | 21.953309800889755 | 23.54240544637044 | 22.637007183498806 | None | None | None | 24.617075602213543 | 29.295965830485024 | 30.244455337524414 | None | 28.837196985880535 | None |

## Slide 3
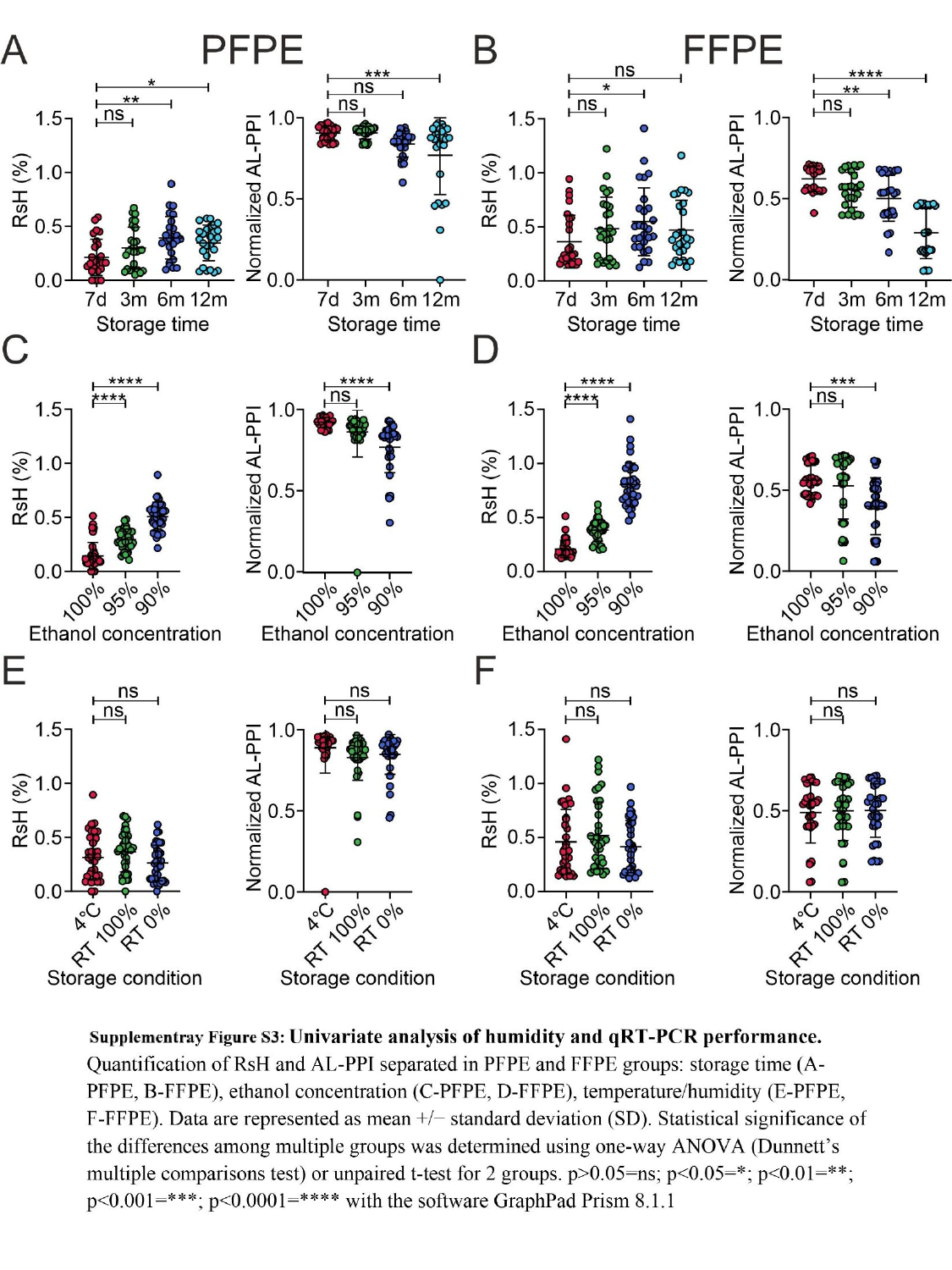

## Slide 4
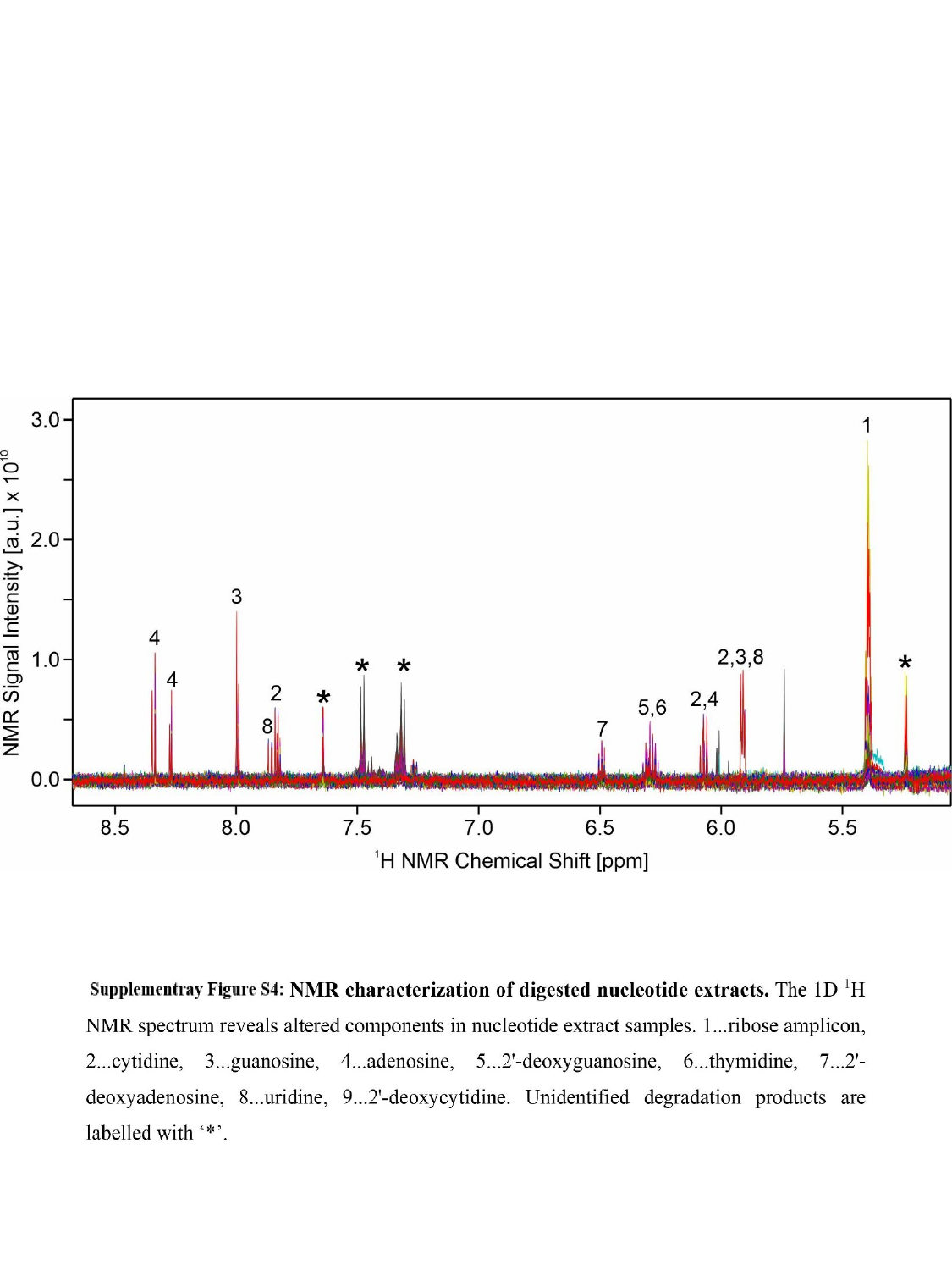

## Slide 5
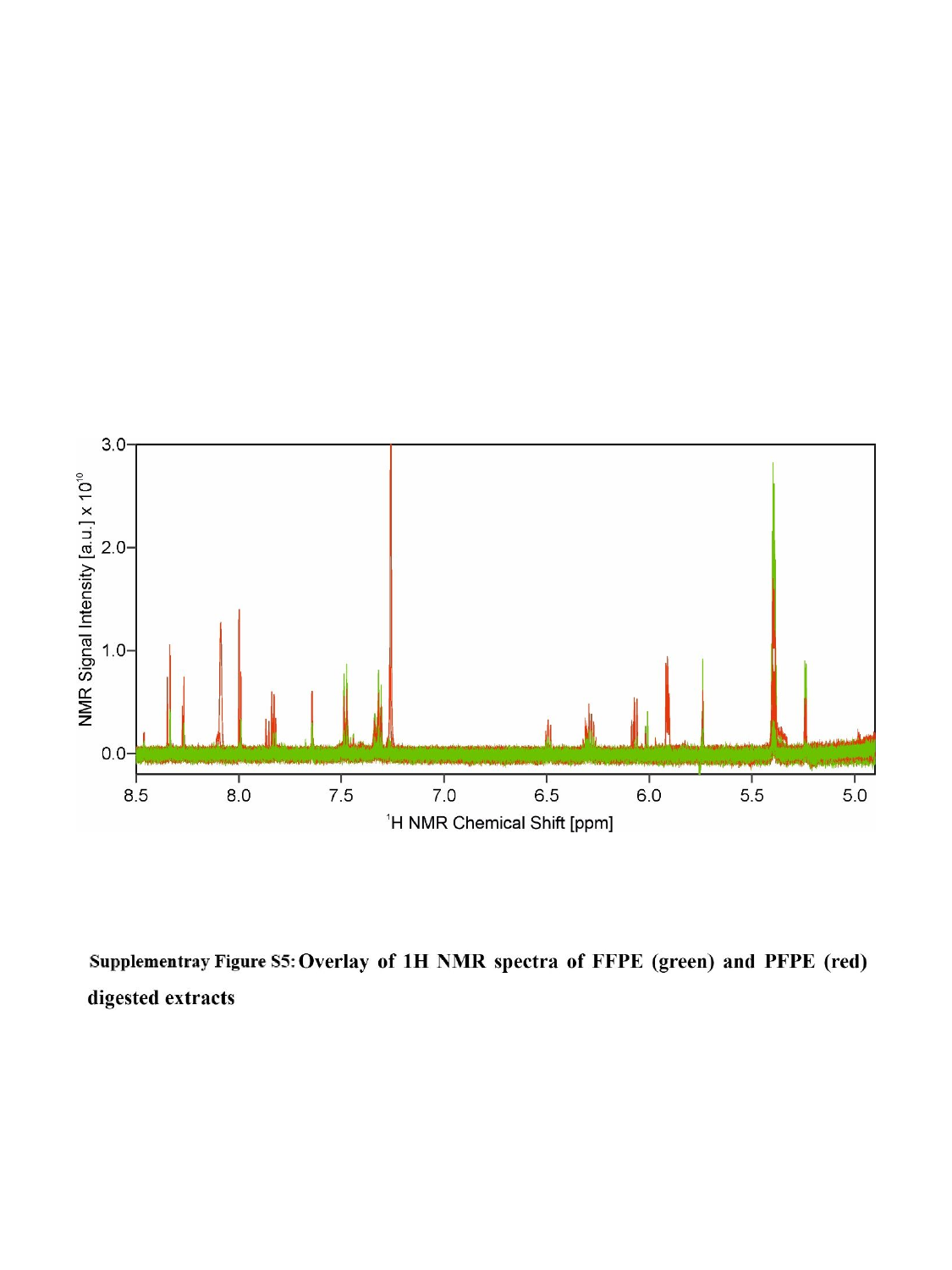

Supplement: Supplementary file 1 [file ijms-24-08010-s001.zip › IJMS Humidity in Fixed Tissue Supplementary Figures .pptx]
