# Supplementary material for: Residual Humidity in Paraffin-Embedded Tissue Reduces Nucleic Acid Stability
Source: Int J Mol Sci. 2023 Apr 28;24(9):8010. doi: 10.3390/ijms24098010 (PMC10178321; doi:10.3390/ijms24098010)
Supplement: Supplementary file 1 [file ijms-24-08010-s001.zip › IJMS Humidity in Fixed Tissue Supplementary Material.pdf]

Residual Humidity in Paraffin Embedded Tissue Reduces Nucleic Acid Stability Supplementary Material

Peter M. Abuja[1*], Daniela Pabst[1], Benjamin Bourgeois[2], Martina Loibner[1], Christine Ulz[1], Iris Kufferath[1], Ulrike Fackelmann[1], Cornelia Stumptner[1], Rainer Kraemer[3], Tobias Madl[2]*, Kurt Zatloukal[1]

[1] Diagnostic & Research Centre for Molecular Biomedicine, Institute of Pathology, Medical University of Graz, Austria.

[2] Gottfried Schatz Research Centre for Cell Signalling, Metabolism and Ageing, Molecular Biology and Biochemistry, Medical University of Graz, Austria and BioTechMed-Graz,Austria

[3] Berghof Products & Instruments GmbH, Eningen, Germany

Supplementary methods

qRT-PCR measurements of *Ywhaz* in mouse liver

RNA was analysed by reverse transcription of 2 μg RNA into cDNA using High-Capacity cDNA Reverse Transcription Kit (ThermoFisher Scientific) according to the manufacturer's instructions. Four microliters of 1:28 cDNA dilutions served as template for qRT-PCR using Luna Universal qPCR Master Mix (New England Biolabs, Ipswich, MA, USA). Fragments of mouse *Ywhaz* with amplicon lengths of 84, 175, 298, 358, 467 and 526 base pairs (bp) were amplified on the QuantStudio 7 Flex qPCR system using the QuantStudio Version 1.3 software (Applied Biosystems, Waltham, MA, USA).

Data points containing nonspecific products, as identified by melting curve analysis, were excluded.

Supplementary Table S1: Sequences, melt temperature and length of the products. For the amplification of the different product lengths of *Ywhaz*, the primer mYwhaz_fw was used as the forward primer and was combined with each one of the other primers (mYwhaz_84, mYwhaz_175, mYwhaz_295, mYwhaz_358, mYwhaz_467 or mYwhaz_526).

Primer name	Gene NCBI NM-Nr.	Primer	Sequence 5'-3'	Tm [°C]	Product length [bp]
mYwhaz_fw	>NM_001253807.1 Mus musculus tyrosine 3-monooxygenase/tryptophan 5-monooxygenase activation protein, zeta polypeptide (Ywhaz), transcript variant 4, mRNA	forward	TCTTACGGCAGAGCGATATGAT	59.18	
mYwhaz_84		reverse	CTCTCCTCATTCGACAGCTCA	59.25	84
mYwhaz_175		reverse	ACCTTCCGTCTTCTGCTCAA	58.95	175
mYwhaz_295		reverse	TTGCGAAGCATTGGGGATCA	60.32	295
mYwhaz_358		reverse	AACCTCGGCCAAGTAACGG	60.00	358
mYwhaz_467		reverse	CCAGTCTGATGGGGTGTGTC	60.04	467
mYwhaz_526		reverse	GGCTTTCTCTGGGGAGTTCA	59.31	526

Analysis of water release from fixed, paraffin-embedded tissue

Supplementary Figure 1 shows a typical water release diagram (black trace) together with the temperature increase (red trace). The temperature programme was empirically chosen to allow separation of adsorbed (<=60 °C) and hydrogen-bonded water (>60°C): adsorbed water was released initially by heating to 40°C for 19 min, then the temperature was raised to 50°C over the next 10 min, to accelerate release of adsorbed water from internal surfaces. Finally, the temperature was raised to 170°C to release hydrogen bonded water, which was essentially complete after another 15 min.

The detected water release thus consists of three overlapping contributions, which were separated using Origin Pro 2.0 software. Since the overlapping water release peaks were extremely asymmetric the automatic deconvolution procedures of Origin Pro 2.0 could not yield satisfactory results. Therefore, integration and separation of the peaks was done semi empirically, as shown in Supplementary Figure 1: first the initial peak was fitted integrated until the end of the first thermal step, and then extrapolated to the end of the measurement, and the baseline to the first peak subtracted. The second peak, beginning with the second thermal step,

was fitted, integrated and the baseline subtracted, then the last peak was fitted and integrated to give the hydrogen-bonded water amount.

Definition and calculation of the performance index for amplicon-length dependent qRT-PCR (AL-PPI)

Highly fragmented RNA and DNA contain fewer long amplicons and thus require more qRT-PCR cycles to reach the cycle of quantitation, C_q. This becomes even more pronounced if the nucleic acids are modified or crosslinked by formalin. While this is immediately obvious from plotting C_q vs. amplicon length (cf. Supplementary Figure 2), it is difficult to use this kind of data for correlation analysis.

However, using several amplicons of increasing length with qRT-PCR reflects NA quality with high sensitivity and resolution, i.e., such an assay can distinguish rather finely between NA of different quality. However, C_q values for amplicons of different lengths are complex to analyse and there might even be no detectable product for longer amplicons. We have therefore parameterized this information into an empirically derived amplicon-length qRT-PCR performance index, AL-PPI that was designed to allow well-graded discrimination between different amplicon-length qRT-PCR assay results.

Supplementary Figure 2 shows a typical series of qRT-PCR results of RNA extracted from mouse liver of different RNA quality. It is immediately obvious that longer amplicons (or fragments) lead to higher C_q values, in particular for FFPE tissue (the decrease of the first three amplicons in the chemically unmodified RNA from PFPE series is possibly due to increased structural stability or better performance during cDNA synthesis of medium-length amplicons over shorter ones). While these C_q series give a good impression of the RNA quality, they cannot be used in statistical analyses, necessitating parameterization in such a way that the parameter appropriately reflects the differences in the curves regarding mean C_q differences and slope towards longer amplicons, and also missing C_q values. Moreover, since C_q is a logarithmic function of the number of amplifiable molecules and we assume that this number decreases linearly with amplicon length, we have decided to take the logarithms of amplicon/amplicon length, or their length difference.

For the smallest amplicon, with length AL_0 and $C_{q,0} > 0$ we define

$$PPI_0 = \frac{\log(aL_0)}{C_{q,0}}.$$

If there is no qRT-PCR product detected, i.e., $C_{q,0} = 0$, then $PPI_0 = 0$.

For all longer amplicons, AL_i (I = 1..n), we define

$$PPI_i = \frac{\log\left(AL_i(AL_i - AL_{i-1})\right)}{C_{q,i}}$$

where the factor (AL_i – AL_{i-1}) serves to spread the index more evenly. Again, if no amplicon is detectable, i.e. $C_{q,i}$ = 0, then PPI_i = 0. The performance index thus becomes lower with increasing C_q (and is reduced maximally for each amplicon that cannot be detected) and increases with the amplicon length and the length difference to the next-shorter amplicon.

AL-PPI is then the sum of all indices

$$AL - PPI = \sum_{i=0}^{n} PPI_i.$$

To facilitate comparison, we normalized all AL-PPI by dividing them by the AL-PPI of freshly cryoconserved tissue, i.e. $AL\text{-}PPI_{cryo} = 1$.

Preparation of nucleic acid samples for NMR analysis

Nucleic acid samples from mouse liver contained impurities of the extraction procedure which were removed by nucleic acid precipitation with ammonium acetate: 100 µL of 10 M ammonium acetate were added to the extracts, followed by addition of 600 µL of isopropanol, and 25 µL of 500 mM $MgCl_2$. Samples were incubated on ice for 1 hour, centrifuged at 4°C and 13,000 rpm for 10 minutes, and supernatants were removed by aspiration using a vacuum pump. For enzymatic digestion of DNA, each pellet was dissolved in 100 µL Tris buffer (pH 7.9, 10 mM), and a reaction mix containing 250 units Benzonase, 0.46 units Phosphodiesterase and 200 units alkaline phosphatase added and incubated overnight at 37°C. After incubation, 50 µL of sample mix were mixed with 500 µL of NMR metabolomics buffer [0.08 M Na_2HPO_4, 5 mM TSP (3-(trimethylsilyl) propionic acid-2,2,3,3-d_4 sodium salt), 0.04 (w/v) % NaN_3 in D_2O, pH adjusted to 7.4 with 8 M HCl and 5 M NaOH] and used for further NMR analysis.

NMR data acquisition and analysis

NMR spectra of digested DNA samples were recorded at 310 K using Bruker Avance Neo 600 MHz NMR spectrometer equipped with a TXI probe head. The Carr–Purcell–Meiboom–Gill (CPMG) pulse sequence was used to acquire ^{1}H 1D NMR spectra with pre-saturation for water suppression (cpmgpr1d, 512 scans, 73728 points in F1, 12019.230 Hz spectral width, 1024 transients, recycle delay 4 s). NMR spectral data were processed as previously described [1]. Briefly, data were processed in Bruker Topspin version 4.0.2 using one-dimensional exponential window multiplication of the FID, Fourier transformation and phase correction. Principal component analysis (PCA), orthogonal partial least squares discriminant analysis (O-PLS-DA) and partial least squares-discriminant analysis (PLS-DA) were performed in Matlab2014b and MetaboAnalyst 4.0 [2] together with all associated data consistency checks as well as cross-validation. In order to validate the statistical significance of the determined differences, the quality assessment statistic Q2 is reported. This measure provides information about cross-validation and is a qualitative measure of consistency between the predicted and original data, with a maximum value of 1. Metabolite identification was carried out using Chenomx NMR Suite 7.6 (Chenomx Inc., Edmonton, AB, Canada). Quantification of metabolites was carried out by signal integration of NMR spectra. To account for different DNA concentrations in the extracts, integrals were normalized based on the amount of extracted DNA. Univariate statistical analysis was carried out using Graph Pad Prism 5.01. (GraphPad Software, La Jolla, CA, USA). Data are represented as mean +/− standard deviation (SD). Statistical differences among multiple groups (one-way ANOVA) are indicated by P-values of < 0.05 (*), < 0.01 (**), or < 0.001 (***).

Supplementary Figure Legends:

Supplementary Figure S1: Water release from FFPE tissue. The temperature profile was empirically designed to allow separation of adsorbed from hydrogen-bonded water (red trace; the oscillations are due to rapid switching of the heating element). At low temperatures (40°C), water trapped in the vial was first released, then water adsorbed at or close to the surface. To speed up expelling adsorbed water from the inner surface of the tissue, the temperature was raised to 50°C. Finally, hydrogen-bonded water was released above 60°C (black trace). For evaluation, the peak shapes (dashed lines are extrapolated of adsorbed water were subtracted

from the total curve, and the remaining third peak integrated, corresponding to amount of hydrogen-bonded water (red area).

Supplementary Figure S2: Amplicon-length dependent qRT-PCR and the AL-PPI

A complete series of qRT-PCR results (6 months storage) is shown for different conditions. The numbers alongside the C_q series are the corresponding AL-PPI.

Supplementary Figure S3: Univariate analysis of humidity and qRT-PCR performance. Quantification of RsH and AL-PPI separated in PFPE and FFPE groups: storage time (A-PFPE, B-FFPE), ethanol concentration (C-PFPE, D-FFPE), temperature/humidity (E-PFPE, F-FFPE). Data are represented as mean +/− standard deviation (SD). Statistical significance of the differences among multiple groups was determined using one-way ANOVA (Dunnett's multiple comparisons test) or unpaired t-test for 2 groups. p>0.05=ns; p<0.05=*; p<0.01=**; p<0.001=***; p<0.0001=**** with the software GraphPad Prism 8.1.1

Supplementary Figure S4: NMR characterization of digested nucleotide extracts. The 1D ^{1}H NMR spectrum reveals altered components in nucleotide extract samples. 1...ribose amplicon, 2...cytidine, 3...guanosine, 4...adenosine, 5...2'-deoxyguanosine, 6...thymidine, 7...2'-deoxyadenosine, 8...uridine, 9...2'-deoxycytidine. Unidentified degradation products are labelled with '*'.

Supplementary Figure S5: Overlay of 1H NMR spectra of FFPE (green) and PFPE (red) digested extracts

1. Stryeck, S.; Horvath, A.; Leber, B.; Stadlbauer, V.; Madl, T. NMR spectroscopy enables simultaneous quantification of carbohydrates for diagnosis of intestinal and gastric permeability. Sci Rep 2018, 8, 14650, doi:10.1038/s41598-018-33104-8.

2. Chong, J.; Soufan, O.; Li, C.; Caraus, I.; Li, S.; Bourque, G.; Wishart, D.S.; Xia, J. MetaboAnalyst 4.0: towards more transparent and integrative metabolomics analysis. Nucleic Acids Res 2018, 46, W486-W494, doi:10.1093/nar/gky310.
